# Methylxanthine Content in Green Tea Supplements: UHPLC Quantification, Method Validation, and Implications for Ergogenic Dosing in Athletes

**DOI:** 10.3390/foods15142504

**Published:** 2026-07-15

**Authors:** Iulia-Alexandra Paliu, Florin-Liviu Gherghina, Andrei Biţă, George Bică, Cornelia Bejenaru, Ludovic Everard Bejenaru, George Dan Mogoşanu, Andrei-Adrian Tica

**Affiliations:** 1 Department of Pharmacology, University of Medicine and Pharmacy of Craiova, 2 Petru Rareş Street, 200349 Craiova, Romania; iulia.paliu@umfcv.ro (I.-A.P.); andrei.tica@umfcv.ro (A.-A.T.); 2 Department of Physical Medicine and Rehabilitation, University of Medicine and Pharmacy of Craiova, 2 Petru Rareş Street, 200349 Craiova, Romania; 3 Drug Research Center, Faculty of Pharmacy, University of Medicine and Pharmacy of Craiova, 2 Petru Rareş Street, 200349 Craiova, Romania; andrei.bita@umfcv.ro (A.B.); cornelia.bejenaru@umfcv.ro (C.B.); ludovic.bejenaru@umfcv.ro (L.E.B.); george.mogosanu@umfcv.ro (G.D.M.); 4 Department of Pharmacognosy & Phytotherapy, Faculty of Pharmacy, University of Medicine and Pharmacy of Craiova, 2 Petru Rareş Street, 200349 Craiova, Romania; 5 Department of Anatomy, University of Medicine and Pharmacy of Craiova, 2 Petru Rareş Street, 200349 Craiova, Romania; 6 Department of Pharmaceutical Botany, Faculty of Pharmacy, University of Medicine and Pharmacy of Craiova, 2 Petru Rareş Street, 200349 Craiova, Romania

**Keywords:** methylxanthines, green tea, UHPLC, quantification, method validation, ergogenic supplements, athletes

## Abstract

Green tea supplements (GTSs) are a favorite among the general population and, due to their methylxanthine (caffeine, theobromine, theophylline) content, might represent a potential ergogenic source in athletes’ performance. The aim of this study was to develop and validate a method that quantifies the methylxanthine content of commercially available GTSs and to evaluate their potential relevance for ergogenic use. An ultra-high-performance liquid chromatography coupled with photodiode array detection and a QDa mass detector (UHPLC–PDA–QDa) method was developed, validated and applied to determine the content of methylxanthines in seven commercially available GTSs. Method validation parameters demonstrated satisfactory performance, with rapid and efficient separation and quantification of the three methylxanthines. Considerable variability was observed among the analyzed products, with caffeine, theobromine, and theophylline contents ranging from 0.37 to 12.13, 0.07 to 3.59, and 0.05 to 0.83 mg/capsule, respectively. However, the methylxanthine amounts measured in the analyzed products were below the doses commonly associated with ergogenic effects. Overall, the developed UHPLC–PDA–QDa method provided a reliable approach for methylxanthine quantification, while the seven products analyzed did not represent meaningful ergogenic sources of these compounds.

## 1. Introduction

Young athletes often struggle to meet their elevated energy demands, especially during competitive seasons, and may find it challenging to make optimal dietary choices that support both growth and performance. As a result, beyond adopting proper nutrition and training programs, many athletes turn to ergogenic aids (defined as external factors designed to enhance performance) as an additional means of gaining a competitive edge. Among these, dietary supplements are widely recognized as nutritional ergogenic aids, particularly those formulated to boost athletic performance and promote faster recovery. However, the growing popularity of these products has surged dramatically in recent years, outpacing the availability of rigorous scientific research necessary to confirm their quality, effectiveness, and, not least, their safety [1,2].

Green tea supplements (GTSs) are commonly used across the globe, with the USA classifying them as the fourth most administered nutritional supplements [3]. Given their polyphenol and methylxanthine content, they are generally recognized as considerably beneficial for athletes’ consumption, with their benefits varying widely. Some benefits consist of their antioxidant role [4], lowering indicators of muscle damage following various forms of exercise [5], enhancing maximal oxygen uptake during cycling exercises performed to exhaustion [6], alleviating neuromuscular parameters such as muscle fatigue and muscle activation [7], and increasing levels of exercise-induced muscle glucose transporter type 4 (GLUT4), a skeletal muscle protein involved in glucose uptake [8].

Nevertheless, when it comes to GTS standardization, the data are inconclusive. First of all, there are no official European specifications for the content of green tea preparations used in nutritional supplements, nor are there any monographs on such preparations in the current edition of the European Pharmacopoeia. The United States Pharmacopoeia provides specifications only for “Powdered Decaffeinated Green Tea Extract”, intended for use in food supplements. Meanwhile, following a request from the European Food Safety Authority (EFSA), the Scientific Panel on Food Additives and Nutrient Sources added to Food (ANS) reviewed only the safety of green tea catechins and recommended that product label should indicate the total catechin content and specify the proportion of epigallocatechin gallate (EGCG), as there were reported cases of liver toxicity associated with it [9].

In addition, current data on the methylxanthine (caffeine, theobromine, and theophylline) content of GTSs are lacking. Considering the recognized physiological and potential ergogenic effects of methylxanthines, particularly relevant to athletic performance, it is essential to establish a clearer understanding and standardization of their concentrations in such products. More specifically, caffeine is considered to be beneficial in enhancing athletes’ performance when administered in doses ranging from 3 to 6 mg/kg, 60 min before exercising, with some of the influenced parameters consisting of the capacity to perform prolonged aerobic activities and exercises of high intensity, and improving physical and cognitive performance in individuals suffering from sleep deprivation [10]. Similarly, even if less investigated, both theophylline and theobromine seem to be valuable in the sports area by influencing parameters such as the endurance of the forearm muscles (300 mg dose of theophylline) or by increasing the speed of running (6 mg/kg dose of theobromine) [11]. On the other hand, anti-doping policies must also be considered: starting in 1984, the International Olympic Committee (IOC) added caffeine to the banned list, and in 2000, it was also banned by the World Anti-Doping Agency (WADA). In 2004, however, the ban was withdrawn by both the IOC and WADA, with the WADA classifying caffeine ever since as a “monitored substance” and still recommending that athletes maintain a urinary threshold below 12 μg/mL, corresponding to less than 10 mg/kg of orally ingested caffeine [10]. The only other methylxanthine that has ever been classified as a prohibited substance due to its potential performance-enhancing effects is theophylline. It appeared on the list of banned doping agents published by the Flemish Government in 1991, with a threshold concentration set at 5 μg/mL in urine. Although the IOC and the WADA have not officially included theophylline on their banned list, scientific research has recommended its consideration. This recommendation was based mostly on the risk of theophylline’s adverse side effects on the subjects. In particular, at a dose of 4.5 mg/kg, most participants in the research experienced nausea and vomiting, underscoring the need to evaluate theophylline for possible inclusion as a banned or restricted substance [11,12].

The analytical determination of methylxanthines has progressed considerably over time, largely due to the structural similarity of these compounds and the complexity of natural samples such as tea, coffee, or cocoa. Early approaches relied on relatively simple techniques like ultraviolet–visible (UV–Vis) spectrophotometry and thin-layer chromatography (TLC), along with extraction procedures that were often time-consuming and involved the use of hazardous reagents, including solvents such as benzene or precipitation methods using lead acetate. Although these classical methods laid important groundwork, they were limited in terms of sensitivity and specificity, particularly when attempting to differentiate closely related dimethylxanthine isomers (theophylline and theobromine) with nearly overlapping spectral characteristics [13,14,15].

As analytical science advanced, high-performance liquid chromatography (HPLC) became the dominant technique for the reliable separation and quantification of these compounds, allowing simultaneous analysis with improved accuracy and reproducibility. More recently, research has shifted toward even more sophisticated platforms such as ultra-high-performance liquid chromatography coupled with mass spectrometry (UHPLC–MS) and micellar electrokinetic capillary chromatography (MECC). These modern methods enable precise structural confirmation through mass-based detection and highly efficient separation mechanisms [14,16,17].

In light of the considerations discussed above, an important question arises: what is the actual content of methylxanthines in commercially available GTSs? Specifically, is the amount present too low to exert physiological effects, or could it potentially exceed safe levels? Given that most available formulations are standardized only based on their catechin content rather than their methylxanthine composition, this study aimed to quantitatively determine the methylxanthine levels in such products. To achieve this, we developed and validated a rapid and cost-efficient ultra-high-performance liquid chromatography with ultraviolet detection (UHPLC–UV) method for the simultaneous analysis of methylxanthines in GTSs.

## 2. Materials and Methods

### 2.1. Chemicals, Reagents, and Standard Solutions

The solvent used in this study included acetonitrile (Merck, Darmstadt, Germany). Ultrapure water was obtained using a HALIOS 6 lab water system (Neptec, Montabaur, Germany) to ensure the required purity for aqueous solutions and dilutions. Formic acid (Merck) was used as an additive to enhance the performance of the mobile phases for UHPLC analysis.

Analytical-grade standards of caffeine, theobromine, and theophylline were purchased from Sigma-Aldrich (Taufkirchen, Germany). To prepare the standard solutions, 5 mg of each compound was accurately weighed and dissolved in a solvent mixture consisting of 95% (*v*/*v*) water and 5% (*v*/*v*) acetonitrile. The resulting solutions were sonicated for 15 min in an ultrasonic bath at 50 °C to ensure complete dissolution. Subsequently, a series of serial dilutions were prepared from these stock solutions to construct the calibration curves.

### 2.2. Green Tea Supplements and Sample Preparation

**Figure 1:**
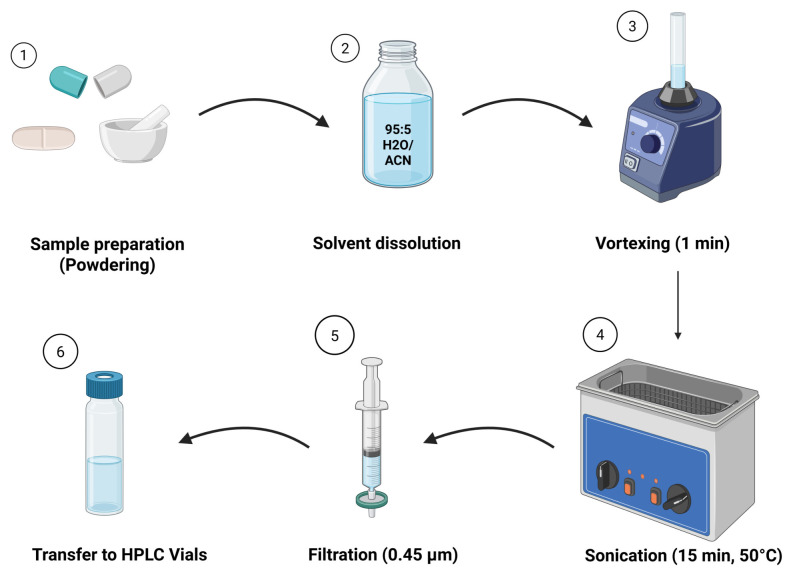
Workflow for preparation of UHPLC samples. ACN: Acetonitrile; H_2_O: Water; HPLC: High-performance liquid chromatography.

Seven GTSs containing green tea extracts were obtained from the commercial nutritional market. According to the information provided on the product labels, the formulations contained green tea (*Camellia sinensis*) leaf powder and/or leaf extract in combination with excipients. The declared amount of green tea-derived material varied among products. For some supplements, the manufacturers indicated that the extracts were standardized in polyphenols, catechins, or EGCG, whereas for others, only the amount of green tea material per dosage unit was reported. Detailed information regarding the extraction solvent, extract ratio, and methylxanthine content was generally not provided by the manufacturers. Six samples were in capsule form, and one was in tablet form. For each supplement, three independent analyses were recorded. The content of one capsule, respectively one tablet, was suspended in 10 mL solvent mixture consisting of 95% (*v*/*v*) water and 5% (*v*/*v*) acetonitrile. The resulting suspension was vortexed for 1 min and subsequently sonicated in an ultrasonic bath at 50 °C for 15 min. The obtained extracts were filtered through 0.45 μm membrane filters to yield clear solutions suitable for chromatographic analysis. The filtered extracts were further diluted, when necessary, with the same water/acetonitrile mixture to ensure that the concentrations of the individual analytes fell within the validated calibration range. The overall dilution factor was calculated as the product of the individual dilution steps. Because caffeine, theobromine, and theophylline occurred at markedly different concentrations in some products, more than one dilution of the same extract was analyzed to quantify each analyte within the validated calibration range. The solutions were transferred into suitable autosampler vials for UHPLC analysis (Figure 1).

### 2.3. UHPLC Analysis

For the analysis of methylxanthine content, we developed a quick and cost-effective method using a UHPLC assay conducted on a Waters Acquity Arc system (Waters, Milford, MA, USA) equipped with a photodiode array (PDA) detector and a QDa mass detector. Separation of the analytes was achieved on a CORTECS C18 column (4.6 × 50 mm, 2.7 μm particle size) maintained at 30 °C. The mobile phase consisted of solvent A (water containing 0.1% formic acid) and solvent C (acetonitrile containing 0.1% formic acid). A gradient elution program was employed, starting with 93% A and 7% C at a flow rate of 0.8 mL/min, followed by a linear change to 30% A and 70% C between 4 and 8 min, which was held constant until 8.00 min. Re-equilibration was performed by returning to 93% A and 7% C at 8.00 min and maintaining this condition until 8.10 min. To ensure reproducibility, the column was equilibrated for 5 min between runs, and all samples were kept at 8 °C before analysis. The volume of injections was 10 μL, and compound quantification was carried out using UV detection at a wavelength of 273 nm. Mass spectrometric confirmation for definitive compound identification was conducted in positive electrospray ionization (ESI) mode, monitoring the protonated molecular ions [M + H]^+^ at *m*/*z* 195 for caffeine, *m*/*z* 181.1 for theophylline, and *m*/*z* 181.1 for theobromine. However, considering that both theophylline and theobromine have the same *m*/*z*, the analytes were identified by comparing the retention times and UV spectral profiles of the sample peaks with those of the corresponding reference standards.

### 2.4. UHPLC Method Validation and Performance Evaluation

The UHPLC method developed for the simultaneous determination of caffeine, theobromine, and theophylline was validated in accordance with International Council for Harmonisation (ICH) guidelines [18]. Method validation covered linearity, accuracy, intermediate precision, and repeatability, with method selectivity inferred from chromatographic performance. System suitability criteria for the proposed UHPLC method were automatically evaluated using Empower version 3.8.0.2 software.

*Linearity* was evaluated over the concentration range of 0.0625–2.00 μg/mL for caffeine, theobromine, and theophylline using six calibration levels, 0.0625 (Level 1), 0.125 (Level 2), 0.250 (Level 3), 0.500 (Level 4), 1.000 (Level 5), and 2.000 μg/mL (Level 6), each analyzed in triplicate, resulting in 18 measurements for each analyte. The volume injection was 10 μL; consequently, the corresponding on-column amounts were 0.625, 1.25, 2.50, 5.00, 10.00, and 20.00 ng, respectively. The calibration equations generated by Empower software were based on peak area as a function of the injected analyte amount (Figures S1–S3). *Accuracy* was assessed at three concentration levels. At each level, three separate solutions were prepared, and each preparation was injected three times, providing nine chromatographic measurements. Percentage recovery was calculated automatically by Empower software relative to the assigned concentration at each validation level, and the overall mean recovery and corresponding percent relative standard deviation (%RSD) were determined. *Repeatability* was assessed by repeated injections (*n* = 3) of standard solutions of caffeine, theobromine, and theophylline at three concentration levels (Levels 2, 4, and 5). *Intermediate precision* of the method was evaluated by analyzing peak areas of caffeine, theobromine, and theophylline over different days. Lower detection limit (LDL) and lower quantification limit (LQL) were estimated from the standard deviation (SD) of the calibration curve intercept and the slope of the corresponding calibration curve.

As an additional assessment, the accuracy of instrumental quantification was evaluated using mixed reference solutions prepared in solvent at three nominal concentration levels (0.10, 0.35, and 1.50 μg/mL) for caffeine, theobromine, and theophylline. The solutions were prepared at each concentration level and analyzed in triplicate. The analyte concentrations were calculated from the corresponding calibration equations using Empower software. Accuracy was expressed as the percentage ratio between the mean experimentally determined concentration and the corresponding nominal concentration. Because the investigated commercial supplements differed in their formulations and excipient compositions, and no analyte-free matrix representative of all products was available, the assessment was performed using reference solutions prepared in solvent.

### 2.5. Statistical Analysis

Statistical analysis of the method validation data was carried out using Empower software (Waters Corporation, Milford, MA, USA). Calibration curves were generated by means of ordinary least-squares linear regression, while the significance of the regression models was evaluated by means of analysis of variance (ANOVA). Regression equation, coefficient of determination (*R*^2^), *F*-value, and associated *p*-value were calculated for each analyte.

The methylxanthine contents determined in the seven commercially available GTS products were statistically evaluated using GraphPad Prism version 8.0.1 (GraphPad Software, San Diego, CA, USA). Differences in caffeine, theobromine, and theophylline contents among the matched GTS products were assessed using the nonparametric Friedman test, followed by Dunn’s post hoc multiple-comparisons test. Because the number of matched products was small, the Friedman test *p*-value was calculated using the exact Friedman distribution. A *p*-value of less than 0.05 was considered statistically significant.

## 3. Results

### 3.1. Validation of the UHPLC Method Through Method Performance Parameters

Linear calibration models were obtained for caffeine, theobromine, and theophylline, with coefficients of determination of at least 0.9998. Regression ANOVA indicated statistically significant calibration models for all three analytes (*p* < 0.0001). The regression equations and the corresponding limits of detection and quantification are summarized in Table 1.

The detection limit was below 0.1 μg/mL for all analytes, while the quantification limit was below 0.3 μg/mL. Mean recoveries were close to 100%, indicating good agreement between the measured and nominal concentrations. Intermediate precision was characterized by %RSD values below 0.6%, while injection repeatability across the three tested concentration levels ranged from 0.13% to 2.56%. The summarized accuracy and precision results are presented in Table 2. Overall, the validation results support the suitability of the method for the quantitative determination of methylxanthines.

In the additional assessment based on back-calculated concentrations, for the accuracy of instrumental quantification, at the nominal concentration of 1.50 μg/mL, the mean experimentally determined concentrations were 1.4855 μg/mL for caffeine, 1.4885 μg/mL for theobromine, and 1.4915 μg/mL for theophylline, corresponding to accuracy values of 99.03%, 99.23%, and 99.43%, respectively. At the intermediate concentration level of 0.35 μg/mL, the mean determined concentrations were 0.3330 μg/mL for caffeine, 0.3340 μg/mL for theobromine, and 0.3345 μg/mL for theophylline, corresponding to accuracy values of 95.14%, 95.43%, and 95.57%, respectively. At the lowest tested concentration of 0.10 μg/mL, mean concentrations of 0.1050 μg/mL for caffeine, 0.1060 μg/mL for theobromine, and 0.1110 μg/mL for theophylline were obtained, resulting in accuracy values of 105.00%, 106.00%, and 111.00%, respectively. However, because 0.10 μg/mL was below the estimated LQL for caffeine (but still in the range of LDL), the corresponding caffeine result was considered exploratory and was not used to support quantitative accuracy within the validated range. Overall, accuracy ranged from 95.14% to 111.00%, with the greatest deviation from the nominal concentration observed for theophylline at the lowest tested level, where the absolute difference between the measured and nominal concentrations was 0.011 μg/mL. The wider relative deviation observed at this level may be attributed to its proximity to the lower end of the calibration range.

### 3.2. Separation of Compounds

The first eluted compound was theobromine, at approximately 1.15 min, followed by theophylline, at approximately 1.68 min. Considering that both theophylline and theobromine have the same *m*/*z*, the analytes were identified by comparing the retention times and UV spectral profiles of the sample peaks with those of the corresponding reference standards. Caffeine was the last compound that was eluted, the timing being registered at approximately 3.25 min and the confirmation being made through agreement of the observed *m*/*z* value with those of the standards. The method demonstrated adequate selectivity, as no interfering peaks were observed at the retention times of caffeine, theobromine, and theophylline.

Representative chromatograms are shown in Figure 2, Figure 3 and Figure 4, while the remaining chromatograms are provided in Figures S4–S8.

### 3.3. Quantification of Methylxanthines in Green Tea Supplements

The validated UHPLC method was applied to the quantitative determination of methylxanthines in commercially available GTSs. Seven different supplement products were analyzed to determine the content of caffeine, theobromine, and theophylline, expressed as mg/capsule. Quantification was performed using the developed method under the optimized chromatographic conditions described above.

Marked differences were observed among the analyzed products. Caffeine was the most abundant methylxanthine in every sample, with a median concentration of 1.653 mg/capsule and individual values ranging from 0.366 to 12.127 mg/capsule. The median theobromine content was 0.155 mg/capsule, with concentrations between 0.070 and 3.587 mg/capsule. Theophylline was present at lower levels overall, ranging from 0.052 to 0.826 mg/capsule, with a median value of 0.119 mg/capsule.

Substantial product-to-product variation was evident for all three compounds. The ratio between the maximum and minimum measured concentrations was approximately 33 for caffeine, 51 for theobromine, and 16 for theophylline. GTS 4 contained the highest amount of each methylxanthine, whereas the other products showed different compositional patterns. For instance, GTS 3 had a comparatively high caffeine content but contained only small amounts of theobromine and theophylline.

**Figure 5:**
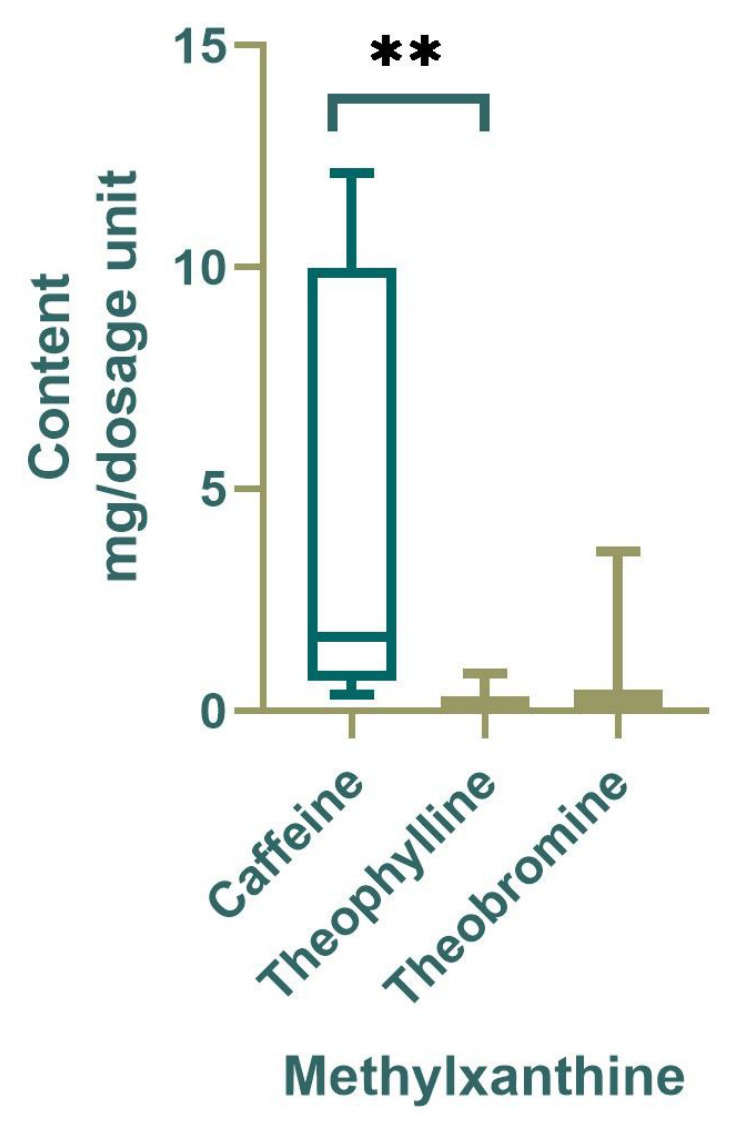
Distribution of caffeine, theobromine, and theophylline contents in the analyzed GTSs. Boxes represent the median and interquartile range, with whiskers indicating the minimum and maximum values. Statistical differences were evaluated using the Friedman test followed by Dunn’s multiple comparisons test. ** Adjusted *p* < 0.01.

When the three methylxanthines were compared across the seven matched products, the Friedman test indicated a significant difference in their content distributions (Friedman statistic: 12.29, *p* = 0.0003). Dunn’s multiple-comparisons test showed a significant difference between caffeine and theophylline (adjusted *p* = 0.0015). The differences between caffeine and theobromine (adjusted *p* = 0.0975) and between theobromine and theophylline (adjusted *p* = 0.5443) were not statistically significant (Figure 5).

Together with the individual product results, this analysis shows that caffeine was consistently the predominant methylxanthine in the investigated GTS samples. Table 3 provides the values of the methylxanthine content in each GTS assessed.

## 4. Discussion

The analytical method developed in the present study enabled the rapid and reliable determination of the three major methylxanthines (caffeine, theobromine, and theophylline) in GTS matrices. To place these findings into context, the analytical performance of the proposed method was compared with previously reported HPLC- and UHPLC-based approaches.

The developed UHPLC–PDA–QDa method enabled the simultaneous determination of the three major methylxanthines, with theobromine, theophylline, and caffeine eluting at approximately 1.15, 1.68, and 3.25 min, respectively. This elution order is consistent with that reported in previous chromatographic studies and reflects the relative polarity of these compounds under reversed-phase conditions [14,16,19,20,21,22]. The complete separation of all three analytes within the first 3.25 min demonstrates that the chromatographic conditions were suitable for their routine determination.

Although the overall method employed gradient elution, all three methylxanthines eluted during the initial isocratic segment, in which the mobile phase was maintained at 93% water containing 0.1% formic acid and 7% acetonitrile containing 0.1% formic acid. The subsequent increase in the proportion of acetonitrile, initiated after 4 min, therefore did not directly influence the retention times of the target analytes. The use of a post-elution gradient was particularly relevant for the analysis of GTSs, as green tea-derived matrices may contain numerous co-extracted phenolic compounds and other constituents with retention behavior distinct from that of the target methylxanthines [23]. This post-elution wash reduces the potential accumulation of matrix components on the stationary phase and prepares the column for subsequent injections.

The retention time of caffeine obtained in the present study was shorter than that reported in directly comparable conventional HPLC methods developed for the simultaneous determination of the same three methylxanthines. Jankech et al. used isocratic elution with water/acetonitrile (90:10, *v*/*v*) and reported retention times of 2.59, 3.66, and 6.60 min for theobromine, theophylline, and caffeine, respectively. Their method employed a 100 × 4.6 mm polar-modified C18 column packed with 3 μm particles, operated at 35 °C and a flow rate of 1.0 mL/min [16]. Similarly, Srdjenovic et al. used a 150 × 4.6 mm C8 column packed with 5 μm particles and an isocratic mobile phase consisting of an aqueous solution containing 0.1% tetrahydrofuran at pH 8 and acetonitrile (90:10, *v*/*v*). At a flow rate of 0.8 mL/min and a column temperature of 25 °C, theobromine, theophylline, and caffeine eluted at approximately 3.25, 4.55, and 7.63 min, respectively [21]. By comparison, the shorter 50 mm CORTECS C18 column packed with 2.7 μm solid-core particles used in the present study allowed all three analytes to elute within 3.25 min under an initial mobile phase composition containing only 7% acetonitrile.

Even shorter retention times have been achieved using highly optimized UHPLC configurations. Zacharis et al. employed isocratic elution with 0.1% formic acid in water and methanol (92.5:7.5, *v*/*v*), together with a 50 × 2.1 mm C18 column packed with 1.7 μm particles, operated at 50 °C and a flow rate of 0.7 mL/min. Under these conditions, the entire separation cycle was completed in less than 3 min, with caffeine eluting as the last methylxanthine at approximately 2.52 min [20]. Aqel et al. achieved baseline separation of theobromine, theophylline, and caffeine at 14, 19, and 28 s, respectively, using isocratic elution with 10% acetonitrile, a 50 × 2.0 mm column packed with 1.8 μm particles, a flow rate of 0.5 mL/min, and a column temperature of 70 °C [14].

These comparisons illustrate that retention time should be interpreted in relation to the complete chromatographic system, including stationary-phase chemistry, column dimensions, particle characteristics, organic modifier and its proportion, mobile-phase additives, flow rate, and operating temperature. Within this context, the present UHPLC method provided a compact analyte elution window using a wider-bore solid-core column under moderate temperature conditions. In addition, it combined PDA-based quantification with QDa-assisted confirmation, while the post-elution gradient facilitated the removal of more strongly retained constituents from the GTS matrices.

Beyond chromatographic separation, the validation results support the suitability of the proposed method for quantitative analysis. The calibration curves showed coefficients of determination ranging from 0.9998 to 0.9999. These results are consistent with those reported for other methods developed for methylxanthine analysis [14,20,24]. Thus, the linear response obtained in the present study is comparable to that of previously validated HPLC- and UHPLC-based procedures.

The method also provided adequate detection and quantification capability, with detection and quantification limits in the sub-μg/mL range. These findings are comparable to those reported for previously validated HPLC- and UHPLC-based procedures for the simultaneous determination of caffeine, theobromine, and theophylline [14,20,24]. Mean recoveries were close to 100%, with minimal bias, while intermediate precision and repeatability generally showed low variability across the tested concentration levels. Overall, the validation parameters were within the ranges reported for methylxanthine analysis in tea and other complex plant-derived matrices, confirming that the method provides reliable quantitative results without requiring more complex tandem mass spectrometric instrumentation.

**Figure 6:**
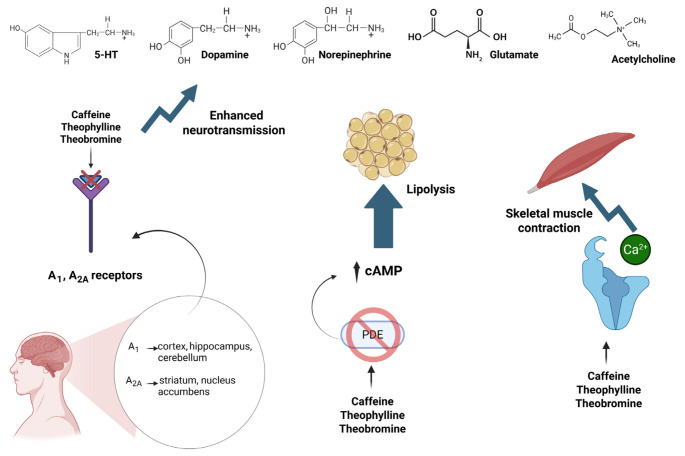
Primary pathways of methylxanthine action relevant to physical exercise. 5-HT: 5-Hydroxytryptamine; A_1_, A_2A_: Adenosine receptors; Ca^2+^: Calcium ions; cAMP: Cyclic adenosine monophosphate; PDE: Phosphodiesterase.

In terms of pharmacological mechanisms, the performance-enhancing effects of methylxanthines are driven by several distinct biological pathways that collectively improve physical output. While these compounds have a broad range of effects, three specific mechanisms are considered the most significant for athletic performance. These processes, ranging from central nervous system (CNS) stimulation to the direct modulation of muscle contraction, work in tandem to boost power and delay the onset of exhaustion. The relationship between these pathways is summarized in Figure 6 and detailed below.

Adenosine is an endogenous compound generated predominantly through the metabolic breakdown of adenosine triphosphate (ATP) and serves diverse physiological roles across multiple organ systems. Within the CNS, it is a key regulator of multiple processes, including neurotransmitter release. Caffeine and theophylline, and to a lesser extent theobromine, inhibit adenosine-mediated signaling by competitively antagonizing multiple receptor subtypes, most notably A_1_ and A_2A_. In the brain, the A_1_ subtype of adenosine receptors is extensively distributed in the hippocampal, cortical, and cerebellar areas, whereas A_2A_ receptors are dispersed to other areas such as the *nucleus accumbens* and *striatum*. Inhibition of A_1_ and A_2A_ adenosine receptors by methylxanthines disrupts adenosinergic control of neuronal function, thereby enhancing neural excitability and promoting the release of multiple neurotransmitters, including noradrenaline, acetylcholine, serotonin, dopamine, and glutamate [25,26,27,28,29,30].

Secondly, by suppressing phosphodiesterase activity, methylxanthines increase intracellular cyclic adenosine monophosphate (cAMP), a vital second messenger for many pathways in the organism. The relevance of this mechanism in terms of athletic performance was demonstrated through increased cAMP concentrations by caffeine in both skeletal muscle and adipose tissue, which activate hormone-sensitive lipases, thereby accelerating lipolysis and promoting the release of free fatty acids and glycerol. Therefore, the enhanced supply of these energy substrates to skeletal muscle reduces the dependence on muscle glycogen during metabolism [15,31].

Beyond these predominant mechanisms, one of the earliest established theories regarding methylxanthine action involves the regulation of intracellular calcium ions (Ca^2+^). Early investigations demonstrated that at high concentrations (ranging from 1 to 10 mM), caffeine alters the way striated muscle handles Ca^2+^ by obstructing its storage within the sarcoplasmic reticulum and promoting its movement across the plasma membrane. Further research has clarified that this effect is largely driven by the sensitization of ryanodine receptors located in both muscular and nervous tissues. By binding to these channels, caffeine acts as a full agonist, facilitating the rapid release of Ca^2+^ and enhancing the sensitivity of myofilaments. However, while caffeine is a highly effective modulator of these channels, theophylline and theobromine are considerably less potent [15,31,32].

While the aforementioned mechanisms provide a theoretical framework for ergo-genic enhancement, in the present study, the quantitative analysis of seven GTSs revealed caffeine levels ranging from a minimum of 0.366 ± 0.0176 mg/capsule to a maximum of 12.127 ± 0.0724 mg/capsule, with concentrations of theobromine and theophylline being even more marginal. The difference between the highest and lowest measured values was approximately 33-fold for caffeine, 51-fold for theobromine, and 16-fold for theophylline. Such variability is consistent with previous analyses of tea-derived products, in which methylxanthine concentrations differed according to tea type, geographical origin, botanical source, extraction procedure, processing conditions, and product formulation [14,16,22,24].

Caffeine was the predominant methylxanthine in every GTS product analyzed in the present study. This distribution agrees with previous investigations of green and black teas, in which caffeine generally occurred at considerably higher concentrations than theobromine and theophylline. In a survey of 83 tea extracts, the average concentrations of caffeine, theobromine, and theophylline were 5561.5, 407.3, and 24.8 mg/kg, respectively [24]. Aqel et al. similarly detected caffeine and theobromine in all 30 commercial tea samples, whereas theophylline was absent from some products [14]. Zacharis et al. [20] detected theophylline in only a limited number of green tea samples, and Jankech et al. [16] did not confirm its presence in the analyzed black and green teas. Attar and Altikatoglu Yapaoz also found substantially lower concentrations of theophylline than of caffeine and theobromine in both green and black tea extracts [22].

In contrast, theophylline was detected in all seven GTS products evaluated in the present study, although its absolute content remained low, ranging from 0.052 to 0.826 mg per dosage unit. This difference may be related to the use of concentrated extracts, differences in raw materials, extraction and manufacturing procedures, or the combination of several ingredients in the finished products. Nevertheless, direct numerical comparison with brewed teas or dried tea leaves is limited because the results are expressed using different units and are based on different sample preparation procedures. The available studies are therefore more useful for comparing the relative distribution and variability of the three methylxanthines than their absolute concentrations.

When these results are compared to the pharmacological thresholds cited in the literature, the levels found in these natural extracts appear remarkably low. Clinical evidence suggests that caffeine’s performance benefits, such as improved aerobic capacity and cognitive resilience, generally require a threshold dose of 3–6 mg/kg [10]. Consequently, reaching a minimum ergogenic dose of 3 mg/kg for a standard 70 kg athlete would require the ingestion of roughly 17 capsules of the supplement with the highest caffeine content, and over 500 capsules of the supplement with the lowest caffeine content. Similarly, theobromine and theophylline have shown efficacy at doses of 6 mg/kg and 300 mg/kg, respectively [11]. Furthermore, while the low levels detected here are insufficient for beneficial physiological responses, they also ensure that the GTSs’ caffeine content remains well below the WADA’s monitored urinary threshold of 12 μg/mL [10]. They also mitigate the risk of adverse effects, such as nausea and vomiting, associated with theophylline at doses of 4.5 mg/kg [11,12].

## 5. Conclusions

In the present study, a rapid and reliable UHPLC method was successfully developed and validated for the simultaneous determination of caffeine, theobromine, and theophylline in GTSs. The method showed excellent linearity, with coefficients of determination of at least 0.9998 and statistically significant regression models for all analytes (*p* < 0.0001). Detection and quantification limits were below 0.3 μg/mL, while the overall mean recoveries calculated using Empower software ranged from 99.66% to 100.04%. Intermediate precision and injection repeatability were also satisfactory, with %RSD values below 0.6% and 2.6%, respectively. Overall, the validation results support the suitability of the method for the quantitative determination of methylxanthines.

Application of the validated method to commercially available supplements revealed considerable variability in methylxanthine content among products. Although all analyzed supplements contain detectable levels of caffeine, theobromine, and theophylline, the absolute amounts per capsule were low and remained well below doses commonly associated with ergogenic effects reported in the literature.

Although these findings cannot be generalized to all commercially available GTSs, they support the need for broader analytical surveillance and more comprehensive re-porting of methylxanthine content. The results also provide useful information for practitioners when evaluating the potential contribution of such supplements to methylxanthine intake.

## Figures and Tables

**Figure 2:**
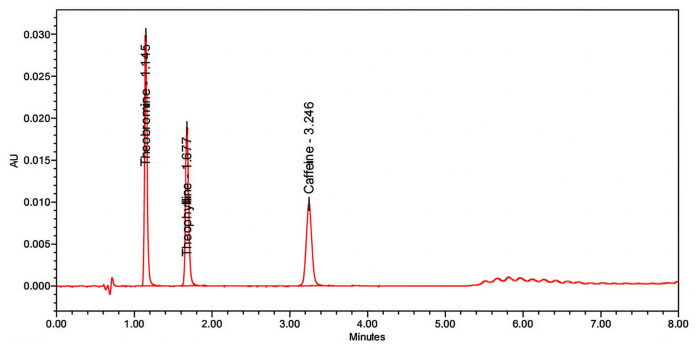
UHPLC chromatogram of analytes in the standard solution. UHPLC: Ultra-high-performance liquid chromatography.

**Figure 3:**
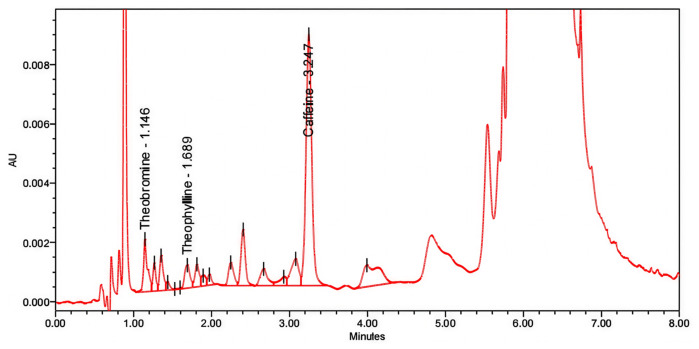
UHPLC chromatogram of analytes in GTS 1. GTS: Green tea supplement.

**Figure 4:**
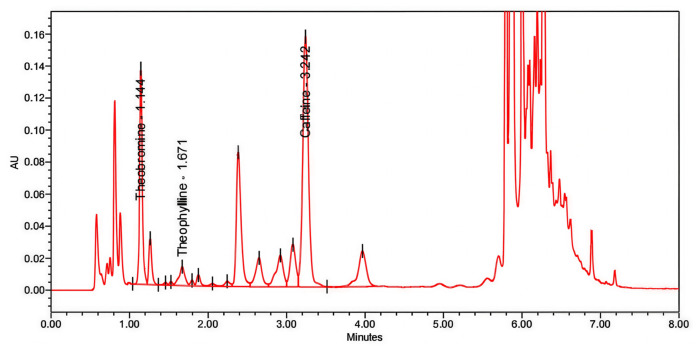
UHPLC chromatogram of analytes in GTS 4.

**Table 1:** Linearity, detection, and quantification parameters of the developed method.

Analyte	Regression Equation	*R* ^2^	LDL (μg/mL)	LQL (μg/mL)
Caffeine	*y* = 3.28 × 10^3^*x* + 7.31 × 10^1^	0.9998	0.09	0.29
Theobromine	*y* = 4.47 × 10^3^*x* − 3.53 × 10^1^	0.9999	0.02	0.07
Theophylline	*y* = 3.59 × 10^3^*x* + 5.67 × 10^1^	0.9999	0.03	0.09

LDL: Lower detection limit; LQL: Lower quantification limit; *R*^2^: Coefficient of determination.

**Table 2:** Comprehensive summary of method validation data.

Validation Parameter	Caffeine	Theobromine	Theophylline
Mean recovery (%)	99.66	100.04	99.67
Accuracy, %RSD	1.23	0.41	0.59
Intermediate precision, %RSD	0.51	0.37	0.27
Repeatability, Level 2, %RSD	2.56	0.40	1.52
Repeatability, Level 4, %RSD	0.73	0.28	0.14
Repeatability, Level 5, %RSD	0.79	0.17	0.1

%RSD: Percent relative standard deviation.

**Table 3:** Quantitative determination of methylxanthines (mean ± SD) in GTSs using the proposed UHPLC method.

Sample	Caffeine Content (mg/capsule)	Theobromine Content (mg/capsule)	Theophylline Content (mg/capsule)
GTS 1	0.665 ± 0.0031	0.070 ± 0.0003	0.052 ± 0.0021
GTS 2	0.366 ± 0.0176	0.131 ± 0.0003	0.061 ± 0.0095
GTS 3	9.991 ± 0.0049	0.155 ± 0.0003	0.054 ± 0.0060
GTS 4	12.127 ± 0.0724	3.587 ± 0.0002	0.826 ± 0.0001
GTS 5	1.653 ± 0.0127	0.361 ± 0.0162	0.128 ± 0.0014
GTS 6	0.724 ± 0.0003	0.102 ± 0.0003	0.332 ± 0.0007
GTS 7	2.045 ± 0.0007	0.481 ± 0.0010	0.119 ± 0.0024

GTS: Green tea supplement; SD: Standard deviation; UHPLC: Ultra-high-performance liquid chromatography.

## Data Availability

The original contributions presented in this study are included in the article/Supplementary Materials. Further inquiries can be directed to the corresponding authors.

## Supplementary Materials

The following supporting information can be downloaded at https://www.mdpi.com/article/10.3390/foods15142504/s1. Figure S1: Calibration curve for caffeine generated using Empower software. The *x*-axis represents the amount of caffeine injected onto the column (0.625–20.0 ng), calculated for an injection volume of 10 μL, while the *y*-axis represents the integrated chromatographic peak area. The central line represents the fitted linear regression, while the adjacent lines represent the corresponding confidence limits generated automatically by Empower software; Figure S2: Calibration curve for theobromine generated using Empower software. The *x*-axis represents the amount of theobromine injected onto the column (0.625–20.0 ng), calculated for an injection volume of 10 μL, while the *y*-axis represents the integrated chromatographic peak area. The fitted regression line and its confidence limits overlap visually because of the narrow confidence interval; Figure S3: Calibration curve for theophylline generated using Empower software. The *x*-axis represents the amount of theophylline injected onto the column (0.625–20.0 ng), calculated for an injection volume of 10 μL, while the *y*-axis represents the integrated chromatographic peak area. The fitted regression line and its confidence limits overlap visually because of the narrow confidence interval; Figure S4: UHPLC chromatogram of analytes in GTS 2. GTS: Green tea supplement; UHPLC: Ultra-high-performance liquid chromatography; Figure S5: UHPLC chromatogram of analytes in GTS 3; Figure S6: UHPLC chromatogram of analytes in GTS 5; Figure S7: UHPLC chromatogram of analytes in GTS 6; Figure S8: UHPLC chromatogram of analytes in GTS 7.

## Abbreviations

The following abbreviations are used in this manuscript:

%RSD	Percent relative standard deviation
95% CI	95% confidence interval
5-HT	5-Hydroxytryptamine
ANOVA	Analysis of variance
ANS	Scientific Panel on Food Additives and Nutrient Sources added to Food
ATP	Adenosine triphosphate
cAMP	Cyclic adenosine monophosphate
CNS	Central nervous system
EFSA	European Food Safety Authority
EGCG	Epigallocatechin gallate
ESI	Electrospray ionization
GLUT4	Glucose transporter type 4
GTS	Green tea supplement
HPLC	High-performance liquid chromatography
ICH	International Council for Harmonisation
IOC	International Olympic Committee
LCL	Lower confidence limit
LDL	Lower detection limit
LQL	Lower quantification limit
MECC	Micellar electrokinetic capillary chromatography
PDA	Photodiode array
PDE	Phosphodiesterase
*R*	Correlation coefficient
*R*^2^	Coefficient of determination
SD	Standard deviation
TLC	Thin-layer chromatography
UCL	Upper confidence limit
UHPLC–MS	Ultra-high-performance liquid chromatography coupled with mass spectrometry
UHPLC–UV	Ultra-high-performance liquid chromatography with ultraviolet detection
UV–Vis	Ultraviolet–visible
WADA	World Anti-Doping Agency

## References

[1] Jovanov P., Đorđić V., Obradović B., Barak O., Pezo L., Marić A., Sakač M. (2019). Prevalence, knowledge and attitudes towards using sports supplements among young athletes. J. Int. Soc. Sports Nutr..

[2] Rodriguez N.R., DiMarco N.M., Langley S. (2009). American Dietetic Association; Dietitians of Canada; American College of Sports Medicine: Nutrition and Athletic Performance. Position of the American Dietetic Association, Dietitians of Canada, and the American College of Sports Medicine: Nutrition and Athletic Performance. J. Am. Diet. Assoc..

[3] Sarma D.N., Barrett M.L., Chavez M.L., Gardiner P., Ko R., Mahady G.B., Marles R.J., Pellicore L.S., Giancaspro G.I., Low Dog T. (2008). Safety of Green Tea Extracts: A Systematic Review by the US Pharmacopeia. Drug Saf..

[4] Hadi A., Pourmasoumi M., Kafeshani M., Karimian J., Maracy M.R., Entezari M.H. (2017). The Effect of Green Tea and Sour Tea (*Hibiscus sabdariffa* L.) Supplementation on Oxidative Stress and Muscle Damage in Athletes. J. Diet. Suppl..

[5] da Silva W., Machado Á.S., Souza M.A., Mello-Carpes P.B., Carpes F.P. (2018). Effect of green tea extract supplementation on exercise-induced delayed onset muscle soreness and muscular damage. Physiol. Behav..

[6] Richards J.C., Lonac M.C., Johnson T.K., Schweder M.M., Bell C. (2010). Epigallocatechin-3-Gallate Increases Maximal Oxygen Uptake in Adult Humans. Med. Sci. Sports Exerc..

[7] Machado Á.S., da Silva W., Souza M.A., Carpes F.P. (2018). Green Tea Extract Preserves Neuromuscular Activation and Muscle Damage Markers in Athletes under Cumulative Fatigue. Front. Physiol..

[8] Tsai T.-W., Chang C.-C., Liao S.-F., Liao Y.-H., Hou C.-W., Tsao J.-P., Cheng I.-S. (2017). Effect of green tea extract supplementation on glycogen replenishment in exercised human skeletal muscle. Br. J. Nutr..

[9] Younes M., Aggett P., Aguilar F., Crebelli R., Dusemund B., Filipič M., Frutos M.J., Galtier P., Gott D., EFSA Panel on Food Additives and Nutrient Sources added to Food (ANS) (2018). Scientific opinion on the safety of green tea catechins. EFSA J..

[10] Guest N.S., VanDusseldorp T.A., Nelson M.T., Grgic J., Schoenfeld B.J., Jenkins N.D.M., Arent S.M., Antonio J., Stout J.R., Trexler E.T. (2021). International Society of Sports Nutrition position stand: Caffeine and exercise performance. J. Int. Soc. Sports Nutr..

[11] Kennedy M. (2021). Effects of theophylline and theobromine on exercise performance and implications for competition sport: A systematic review. Drug Test. Anal..

[12] Pigozzi F., Sacchetti M., Di Salvo V., Alabiso A., Fagnani F., Parisi A. (2003). Oral theophylline supplementation and high-intensity intermittent exercise. J. Sports Med. Phys. Fit..

[13] Komes D., Horžić D., Belščak A., Kovačević Ganič K., Baljak A. (2009). Determination of Caffeine Content in Tea and Maté Tea by using Different Methods. Czech J. Food Sci..

[14] Aqel A., Almulla A., Al-Rifai A., Wabaidur S.M., ALOthman Z.A., Badjah-Hadj-Ahmed A.-Y. (2019). Rapid and Sensitive Determination of Methylxanthines in Commercial Brands of Tea Using Ultra-High-Performance Liquid Chromatography-Mass Spectrometry. Int. J. Anal. Chem..

[15] Monteiro J.P., Alves M.G., Oliveira P.F., Silva B.M. (2016). Structure-Bioactivity Relationships of Methylxanthines: Trying to Make Sense of All the Promises and the Drawbacks. Molecules.

[16] Jankech T., Maliarová M., Martinka N. (2019). Determination of methylxanthines in tea samples by HPLC method. Nova Biotechnol. Chim..

[17] Pomilio A.B., Trajtemberg S.P., Vitale A.A. (2005). Micellar electrokinetic capillary chromatography of methylxanthines-containing beverages: Discussion of the molecular species involved. J. Sci. Food Agric..

[18] European Medicines Agency (EMA), Committee for Medicinal Products for Human Use (2023). ICH Q2(R2) Guideline on validation of analytical procedures—Step 5—Revision 2. Reference No. EMA/CHMP/ICH/82072/2006. https://www.ema.europa.eu/en/ich-q2r2-validation-analytical-procedures-scientific-guideline.

[19] Tzanavaras P.D., Themelis D.G. (2007). Development and validation of a high-throughput high-performance liquid chromatographic assay for the determination of caffeine in food samples using a monolithic column. Anal. Chim. Acta.

[20] Zacharis C.K., Kika F.S., Tzanavaras P.D., Fytianos K. (2013). Development and validation of a rapid ultra high pressure liquid chromatographic method for the determination of methylxanthines in herbal infusions. J. Chromatogr. B.

[21] Srdjenovic B., Djordjevic-Milic V., Grujic N., Injac R., Lepojevic Z. (2008). Simultaneous HPLC Determination of Caffeine, Theobromine, and Theophylline in Food, Drinks, and Herbal Products. J. Chromatogr. Sci..

[22] Attar A., Altikatoglu Yapaoz M. (2023). The analysis of methylxanthine fractions obtained from *Camellia sinensis* cultivated in Turkey and effects on the in vitro inhibition of CYP2D6 enzyme. Biotechnol. Appl. Biochem..

[23] Bae I.K., Ham H.M., Jeong M.H., Kim D.H., Kim H.J. (2015). Simultaneous determination of 15 phenolic compounds and caffeine in teas and mate using RP-HPLC/UV detection: Method development and optimization of extraction process. Food Chem..

[24] Baek G.-H., Yang S.-W., Yun C.-I., Lee J.-G., Kim Y.-J. (2022). Determination of methylxanthine contents and risk characterisation for various types of tea in Korea. Food Control.

[25] Sheth S., Brito R., Mukherjea D., Rybak L.P., Ramkumar V. (2014). Adenosine Receptors: Expression, Function and Regulation. Int. J. Mol. Sci..

[26] Müller C.E., Jacobson K.A., Fredholm B.B. (2011). Xanthines as Adenosine Receptor Antagonists. Methylxanthines.

[27] Chou C.-C., Vickroy T.W. (2003). Antagonism of adenosine receptors by caffeine and caffeine metabolites in equine forebrain tissues. Am. J. Vet. Res..

[28] Tchekalarova J.D., Kubová H., Mareš P. (2014). Early caffeine exposure: Transient and long-term consequences on brain excitability. Brain Res. Bull..

[29] Lazarus M., Shen H.-Y., Cherasse Y., Qu W.-M., Huang Z.-L., Bass C.E., Winsky-Sommerer R., Semba K., Fredholm B.B., Boison D. (2011). Arousal Effect of Caffeine Depends on Adenosine A2A Receptors in the Shell of the Nucleus Accumbens. J. Neurosci..

[30] Schmidt A.P., Lara D.R., Souza D.O. (2007). Proposal of a guanine-based purinergic system in the mammalian central nervous system. Pharmacol. Ther..

[31] Institute of Medicine (US) Committee on Military Nutrition Research (2001). Caffeine for the Sustainment of Mental Task Performance: Formulations for Military Operations.

[32] Donoso P., O’Neill S.C., Dilly K.W., Negretti N., Eisner D.A. (1994). Comparison of the effects of caffeine and other methylxanthines on [Ca2+]i in rat ventricular myocytes. Br. J. Pharmacol..

