# Supplementary material for: Methylxanthine Content in Green Tea Supplements: UHPLC Quantification, Method Validation, and Implications for Ergogenic Dosing in Athletes
Source: Foods. 2026 Jul 15;15(14):2504. doi: 10.3390/foods15142504 (PMC13409502; doi:10.3390/foods15142504)
Supplement: Supplementary file 1 [file foods-15-02504-s001.zip › foods-4378439-supplementary.pdf]

Supplementary Material

Article

Methylxanthine Content in Green Tea Supplements: UHPLC Quantification, Method Validation, and Implications for Ergogenic Dosing in Athletes

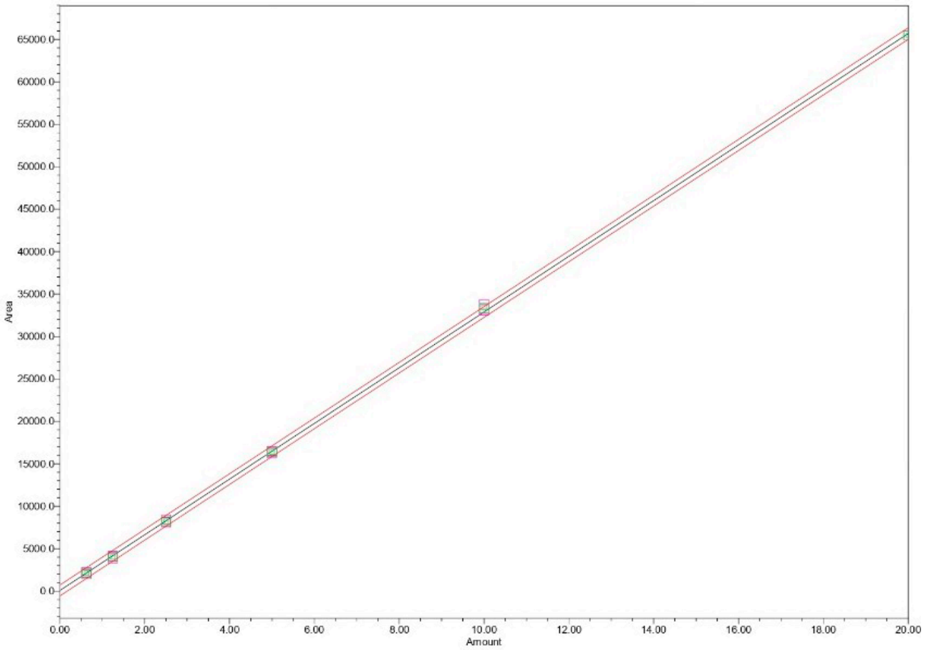

Figure S1. Calibration curve for caffeine generated using Empower software. The x-axis represents the amount of caffeine injected onto the column (0.625–20.0 ng), calculated for an injection volume of 10 μL, while the y-axis represents the integrated chromatographic peak area. The central line represents the fitted linear regression, while the adjacent lines represent the corresponding confidence limits generated automatically by Empower software.

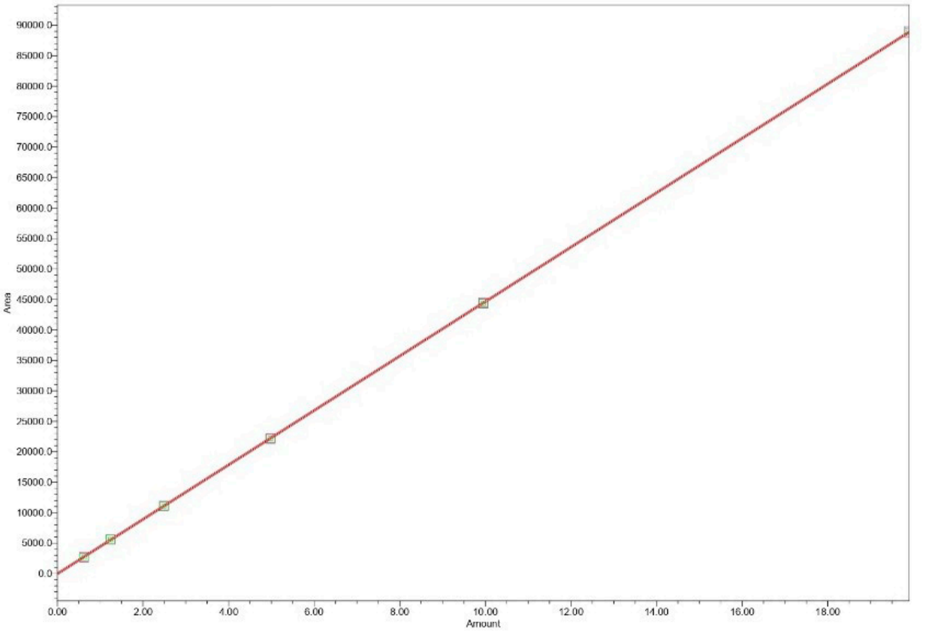

Figure S2. Calibration curve for theobromine generated using Empower software. The x-axis represents the amount of theobromine injected onto the column (0.625–20.0 ng), calculated for an injection volume of 10 μL, while the y-axis represents the integrated chromatographic peak area. The fitted regression line and its confidence limits overlap visually because of the narrow confidence interval.

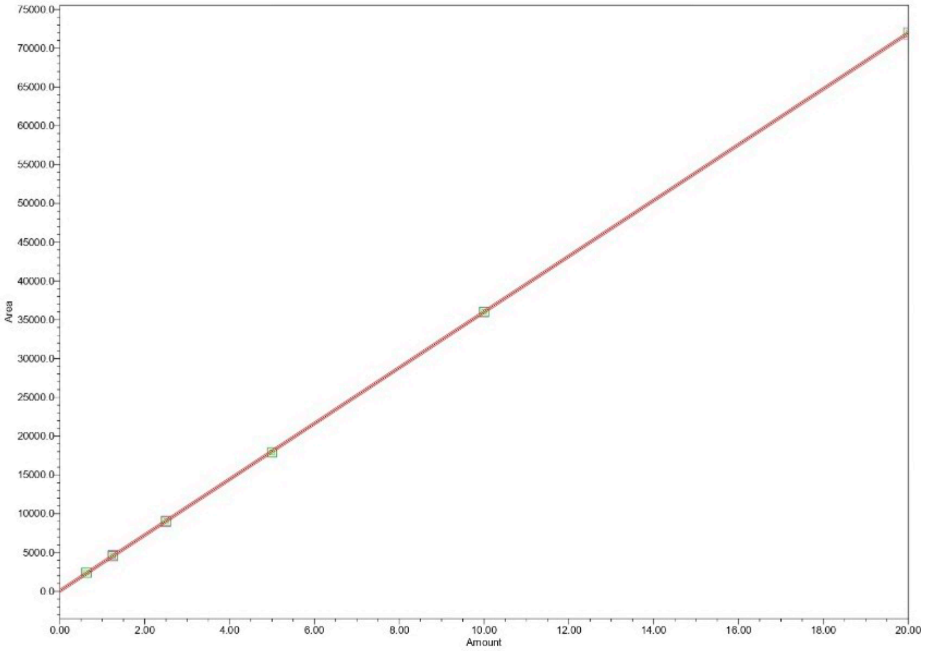

Figure S3. Calibration curve for theophylline generated using Empower software. The x-axis represents the amount of theophylline injected onto the column (0.625–20.0 ng), calculated for an injection volume of 10 μL, while the y-axis represents the integrated chromatographic peak area. The fitted regression line and its confidence limits overlap visually because of the narrow confidence interval.

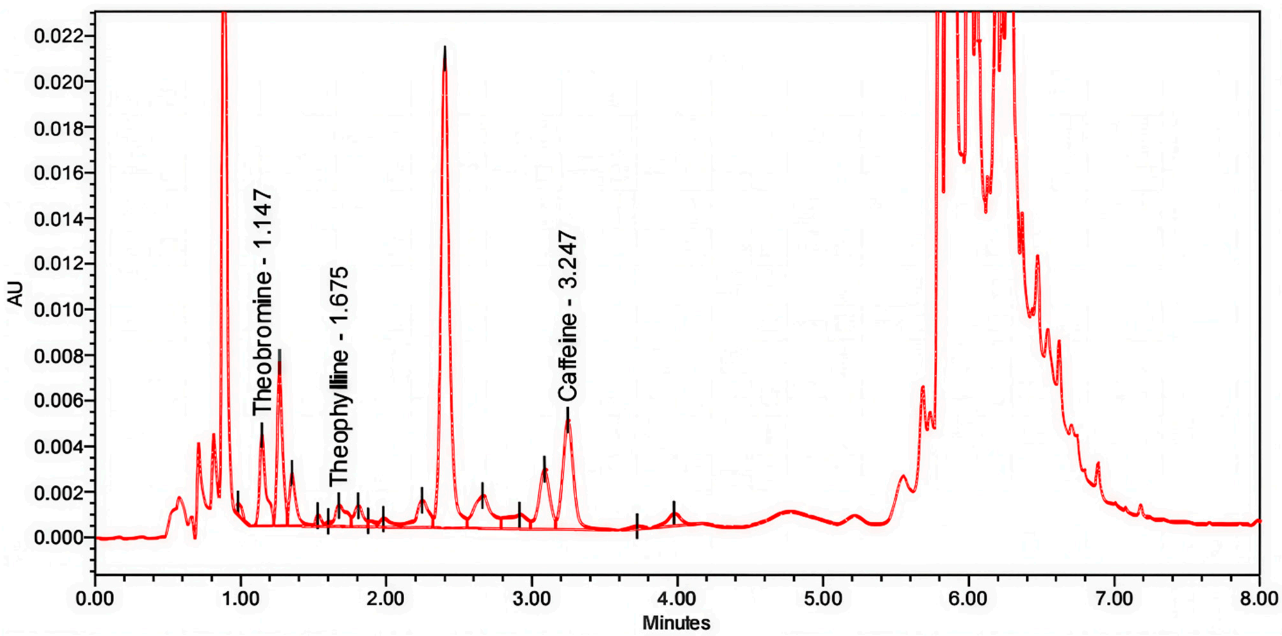

Figure S4. UHPLC chromatogram of analytes in GTS 2. GTS: Green tea supplement; UHPLC: Ultra-high-performance liquid chromatography.

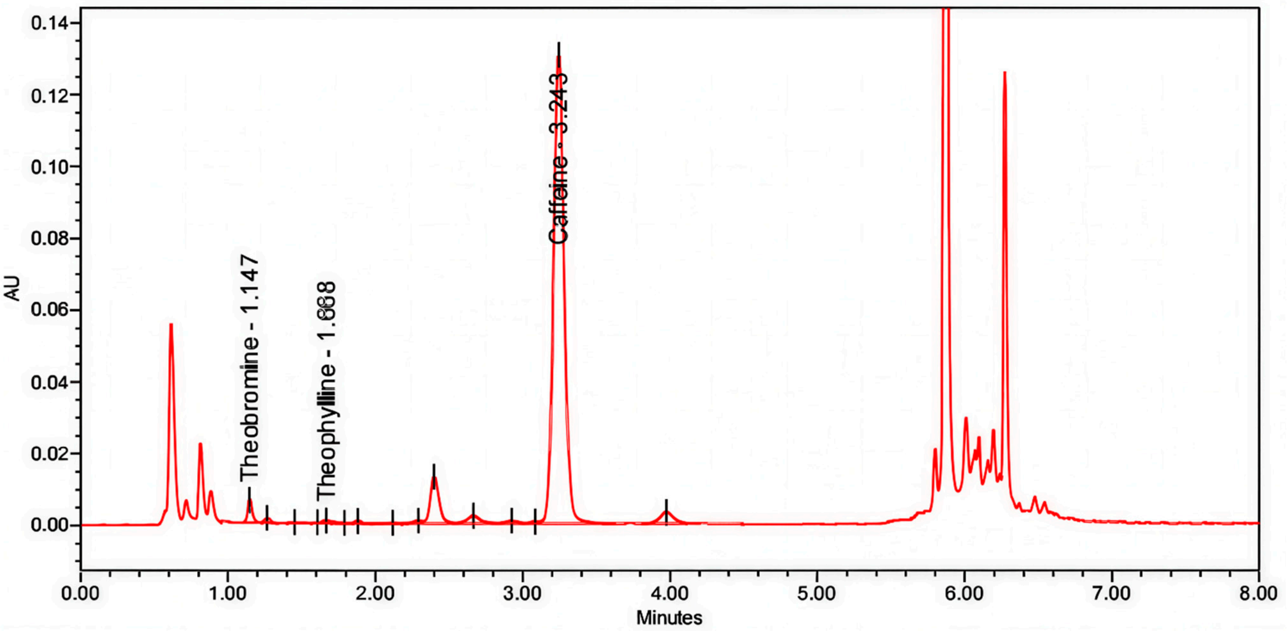

Figure S5. UHPLC chromatogram of analytes in GTS 3.

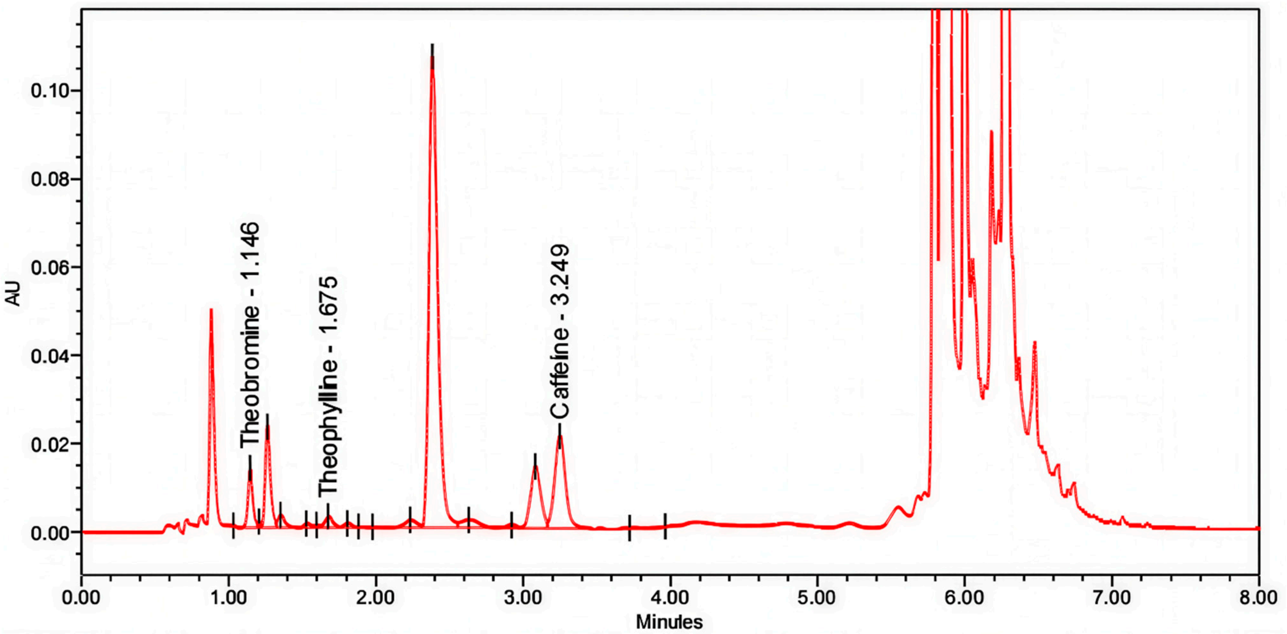

Figure S6. UHPLC chromatogram of analytes in GTS 5.

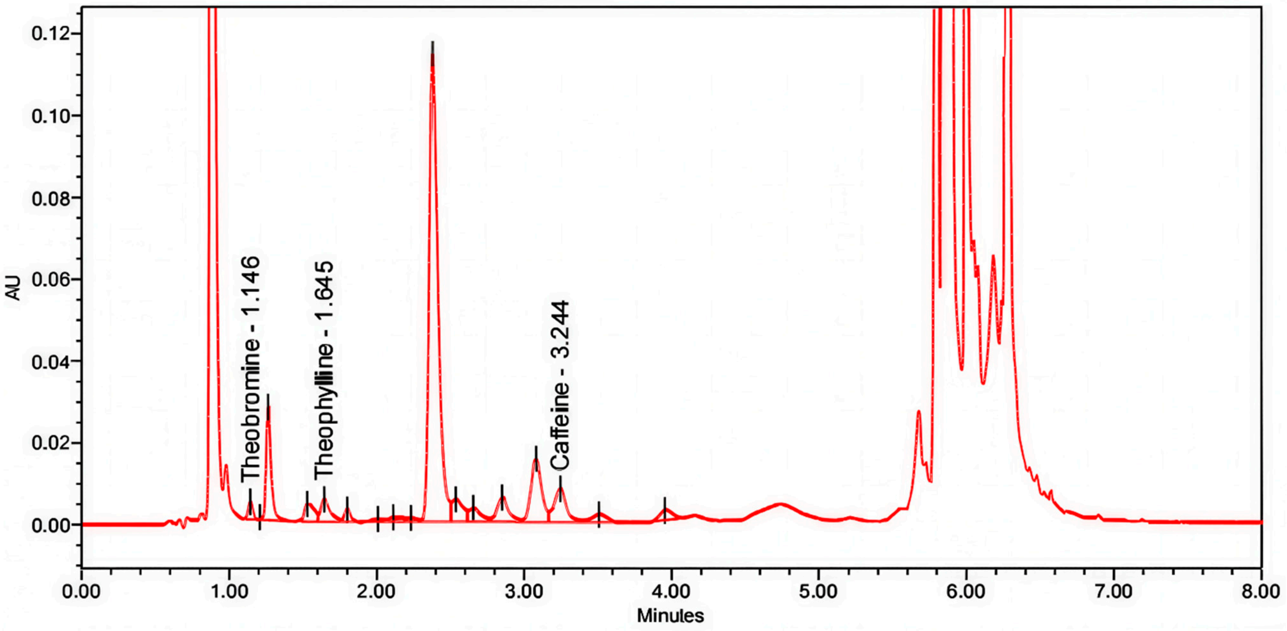

Figure S7. UHPLC chromatogram of analytes in GTS 6.

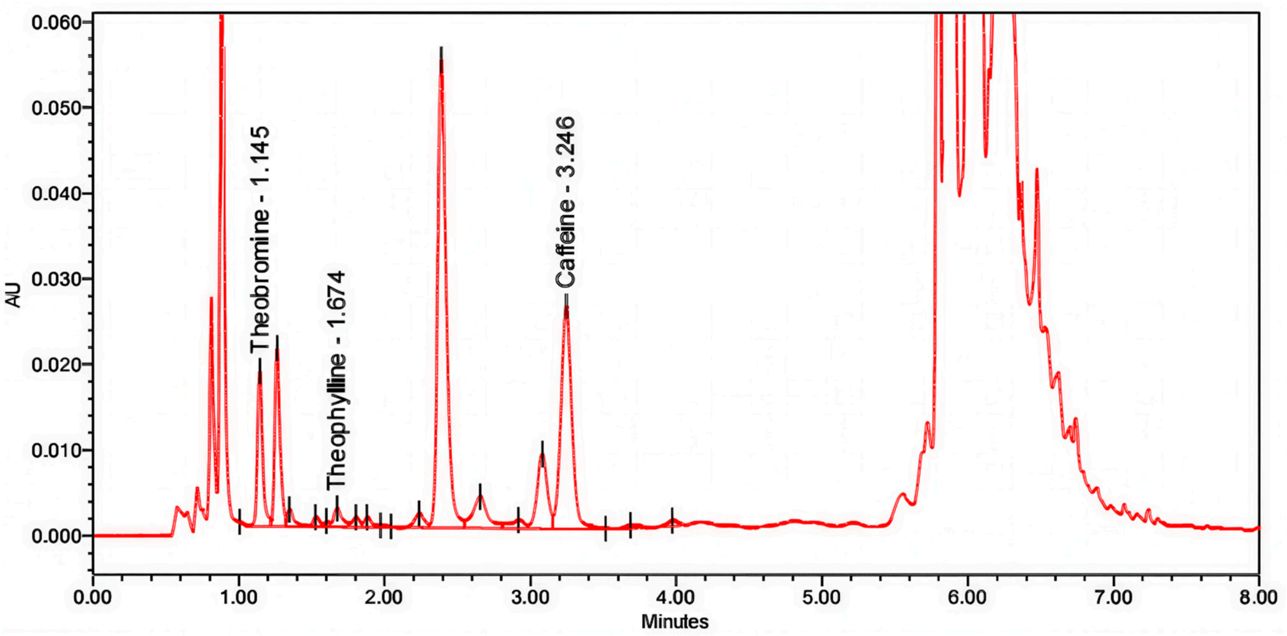

Figure S8. UHPLC chromatogram of analytes in GTS 7.
